# Staff meeting effectiveness and team performance among nurses and midwives in a tertiary hospital in Qatar

**DOI:** 10.3389/fpsyg.2026.1917610

**Published:** 2026-09-23

**Authors:** Parwaneh Elias Shibani, Charles Wesley Devanesan, Aseel Hatamleh, Yahya Al Jarah, Kalpana Singh

**Affiliations:** 1 Administrative Service, Hamad Medical Corporation, Doha, Qatar; 2 Nursing and Inpatient Service, Hamad Medical Corporation, Doha, Qatar; 3 Research Department, Hamad Medical Corporation, Doha, Qatar

**Keywords:** healthcare management, meeting effectiveness, nursing workforce, occupational health, organizational culture, team performance

## Abstract

Staff meetings are routine but often underused tools for workforce coordination in healthcare. This brief research report assessed perceived staff meeting effectiveness, team performance, and related organizational conditions among registered nurses and midwives in a tertiary hospital in Qatar. A cross-sectional correlational survey was completed by 190 eligible staff using the Staff Meeting Effectiveness Questionnaire, the Team Performance Scale, and organizational-context items. Meeting effectiveness and team performance were generally favorable and strongly associated (Spearman rho = 0.764, *p* < 0.001). In bivariate analyses, access to professional development, access to resources, leadership communication, and perceived organizational culture were each associated with both outcomes (all *p* < 0.001). In multivariable regression, organizational culture was the most consistent independent factor associated with both meeting effectiveness and team performance. These findings suggest that routine meetings should be managed as part of the healthcare work environment rather than as administrative activities. Practical priorities include clear meeting purpose, structured agendas, action tracking, and consistent leadership follow-up.

## Introduction

Meetings are a routine feature of health service work, but they are often treated as an administrative necessity rather than a management process that can influence workforce coordination. In hospitals, staff meetings provide a recurring forum for sharing information, clarifying priorities, discussing service constraints, reviewing quality issues, and agreeing follow-up actions. When poorly prepared or weakly facilitated, meetings may consume scarce clinical time without improving shared understanding. When structured well, they may support communication, participation, and coordinated action across complex clinical environments (Allen and Rogelberg, 2018; Král et al., 2023).

For nursing and midwifery services, meetings sit at the intersection of frontline care, staff engagement, occupational wellbeing, and local service governance. They are one of the few regular spaces where workforce concerns, resource constraints, practice changes, quality priorities, and patient-flow pressures can be discussed with staff who deliver care directly. This makes meeting quality relevant to public health system performance: poor communication and weak follow-up can increase frustration and disengagement, while clear and inclusive meetings may strengthen psychological safety, role clarity, and shared accountability.

This issue is especially important in large multinational hospital workforces, where communication practices, expectations of leadership, and professional experiences may vary across cultural and clinical groups. Evidence from teamwork and leadership research suggests that communication quality, leader behavior, shared understanding, and team climate are associated with team effectiveness, safety, and staff engagement (Rosen et al., 2018; Buljac-Samardzic et al., 2020; Scott et al., 2022). However, empirical work directly linking staff meeting effectiveness with team performance in nursing settings remains limited, particularly in Middle Eastern health systems.

This brief research report examined perceived staff meeting effectiveness and team performance among registered nurses and midwives in a tertiary hospital in Qatar. It also explored whether these outcomes were associated with modifiable organizational conditions, including professional development, resource access, leadership communication, and perceived organizational culture. The aim was to generate practical evidence for managers seeking low-cost ways to strengthen workforce coordination, team functioning, and the everyday work environment.

The study is framed as an occupational health and public health management issue because the functioning of clinical teams affects workforce sustainability, communication reliability, and the capacity of services to respond to operational risk. Rather than focusing only on formal staffing levels or large-scale reforms, the report examines a routine process that managers can assess and improve within existing resources. This focus is relevant to hospitals seeking practical approaches that support both staff experience and service delivery.

## Method

Design and setting. A cross-sectional descriptive correlational survey was conducted in a tertiary hospital in Qatar. Clinical areas represented in the sample included medicine, surgery, emergency care, maternity and obstetrics, pediatrics, intensive care, outpatient services, and nursing administration. The meetings of interest were routine staff meetings and related unit forums used for clinical and operational coordination, including unit meetings, councils, leadership forums, and other administrative or multidisciplinary meetings relevant to service delivery.

Participants. Eligible participants were registered nurses and midwives employed for at least 1 year who attended at least 75% of unit staff meetings. Staff on extended leave during data collection or deployed to another unit for more than 3 months were excluded. These criteria were selected to ensure that respondents had sufficient exposure to the meeting processes being assessed. The approximate eligible workforce was 450 nurses and midwives. *A priori* power analysis for detecting a medium association (*r* = 0.30; alpha = 0.05; power = 0.80) indicated a minimum sample of about 157; the target was inflated to 174 to allow for incomplete responses (Cohen, 2013). A total of 190 participants completed the survey.

Measures. Meeting effectiveness was assessed using the Staff Meeting Effectiveness Questionnaire, a 42-item validated measure covering domains such as facilitation, information sharing, participation, structure, and follow-up (Bélisle et al., 2022). Items are rated on a 5-point Likert scale, with higher scores indicating more effective meetings. Team performance during meetings was assessed using the Team Performance Scale, an 18-item tool scored from 0 to 6 that captures participation, respect, task focus, problem solving, conflict management, and other behaviors relevant to meeting-based teamwork (Thompson et al., 2009).

Additional demographic and organizational-context items were collected to characterize respondents and examine conditions that may shape meeting experiences. These included age, gender, education, years of nursing experience, clinical area, shift pattern, weekly working hours, meeting frequency and duration, and role during meetings. Organizational variables assessed perceived access to professional development, access to resources such as staffing and equipment, communication with leaders, and perceived organizational culture. These variables were included because they represent modifiable conditions that managers can influence more directly than fixed demographic factors.

Data collection and bias reduction. Invitation emails containing an electronic survey link were sent to eligible staff, and two reminders were issued over approximately 6 weeks. Participation was voluntary and anonymous, and no personal identifiers were collected. The use of validated instruments, anonymous responses, and electronic completion was intended to reduce information bias and social desirability effects, particularly because the survey asked staff to rate meetings, leadership communication, and organizational conditions. Reasons for non-participation were not collected because the survey was voluntary and anonymous.

Data were analyzed using Stata version 17. Descriptive statistics summarized participant characteristics. Categorical variables were reported as frequencies and percentages. Continuous scores were summarized using means and standard deviations or medians and interquartile ranges, as appropriate. Because outcome variables were not normally distributed, Mann–Whitney U tests and Kruskal-Wallis tests were used for group comparisons, and Spearman correlation assessed the association between total meeting effectiveness and total team performance.

Multivariable linear regression examined factors independently associated with meeting effectiveness and team performance. Variables considered theoretically relevant or managerially important were entered into the models, including marital status, years of nursing experience, professional development, resource access, communication with leaders, and organizational culture perception. Regression coefficients, 95% confidence intervals (CIs), and *p*-values were reported. Complete case analysis was used, and statistical significance was set at *p* < 0.05.

Ethics. The study was approved as exempt by the Medical Research Center, Hamad Medical Corporation, Doha, Qatar (Protocol No. MRC-01-25-731). Completion and submission of the anonymous survey constituted implied informed consent.

The analysis was intentionally focused because the Brief Research Report format required concise presentation of the most relevant associations. Detailed descriptive and bivariate findings were therefore summarized in the main manuscript and supported by a Supplementary Table S1. This approach preserved transparency while keeping the primary narrative centered on the relationship between meeting effectiveness, team performance, and modifiable organizational conditions.

## Results

The final sample comprised 190 respondents (Table 1). Most participants were aged 31–40 years (58.4%), female (76.8%), married (87.9%), and held a bachelor’s degree (74.7%). Most reported 11–20 years of nursing experience, worked 8-h shifts, and attended staff meetings monthly. The workforce was multinational, with Indian, Filipino, and Jordanian staff forming the largest nationality groups. This diversity is operationally relevant because structured meeting processes may help create consistent expectations across professional and cultural backgrounds.

**Table 1:** Selected participant and organizational characteristics (*N* = 190).

Characteristic	Summary
Age	31–40 years: 111 (58.4%); 41–50 years: 57 (30.0%); > = 51 years: 17 (8.9%); 21–30 years: 5 (2.6%)
Gender	Female: 146 (76.8%); male: 44 (23.2%)
Marital status	Married: 167 (87.9%); single: 20 (10.5%); divorced/widowed: 3 (1.6%)
Education	Bachelor’s: 142 (74.7%); master’s: 30 (15.8%); diploma/doctoral: 18 (9.5%)
Nursing experience	11–15 years: 70 (36.8%); 16–20 years: 52 (27.4%); > = 21 years: 37 (19.5%); <=10 years: 31 (16.3%)
Nationality region	Non-Arab: 119 (62.6%); Arab: 71 (37.4%)
Largest clinical areas	Pediatrics: 38 (20.0%); obstetrics/gynecology: 38 (20.0%); surgery/OT: 34 (17.9%); pediatric long-term care: 22 (11.6%)
Shift and hours	8-h shifts: 176 (92.6%); 40 h/week: 164 (86.3%)
Meeting attendance	Monthly: 179 (94.2%); weekly: 10 (5.3%); quarterly: 1 (0.5%)

Overall perceptions of meeting effectiveness and team performance were favorable (Table 2). Meeting effectiveness had a mean score of 145.0 (SD 20.6) and a median of 147.5 (IQR 131.0–156.0). Team performance had a mean score of 62.6 (SD 14.1) and a median of 65.0 (IQR 55.0–71.0). Most participants rated their teams as either working well (52.1%) or doing a great job (41.6%). Meeting effectiveness and team performance were strongly associated (Spearman rho = 0.764, *p* < 0.001), indicating that staff who perceived meetings as more effective also tended to report stronger meeting-related team functioning.

**Table 2:** Outcome scores and key bivariate findings.

Item	Finding
Meeting effectiveness score	Mean 145.0 (SD 20.6); median 147.5 (IQR 131.0–156.0)
Team performance score	Mean 62.6 (SD 14.1); median 65.0 (IQR 55.0–71.0)
Team performance level	Needs improvement: 12 (6.3%); working well: 99 (52.1%); great job: 79 (41.6%)
Correlation between outcomes	Spearman rho = 0.764, *p* < 0.001
Significant bivariate organizational factors	Professional development, resources, leader communication, and organizational culture were each associated with both outcomes (all *p* < 0.001).

Most demographic and meeting-logistic variables were not significantly associated with both outcomes. Age, gender, nationality region, shift type, meeting frequency, meeting duration, and meeting role did not show statistically significant differences in meeting effectiveness or team performance. Meeting effectiveness differed by marital status (*p* = 0.035) and years of nursing experience (*p* = 0.005), but corresponding differences in team performance were not statistically significant.

The clearest bivariate findings concerned organizational conditions. Access to professional development, access to resources, communication with leaders, and perceived organizational culture were each significantly associated with both meeting effectiveness and team performance (all *p* < 0.001). Higher ratings of leadership communication and organizational culture showed clear gradients, with higher median scores for both outcomes among staff reporting more positive organizational conditions. This suggests that meeting quality may be experienced as part of a wider work environment rather than as a stand-alone event.

In multivariable regression (Table 3), organizational culture perception was the most consistent independent factor associated with both outcomes. Compared with respondents reporting very low organizational culture perception, those reporting high and very high organizational culture perception had significantly higher meeting-effectiveness scores and team-performance scores. Staff with 11–15 years of nursing experience had significantly lower meeting-effectiveness scores than those with 1–5 years of experience (beta = −16.93, 95% CI: −33.21 to −0.64, *p* = 0.042). Marital status, professional development, resource access, and leadership communication were not statistically significant in the adjusted models.

**Table 3:** Selected adjusted estimates from multivariable linear regression models.

Variable/domain	Meeting effectiveness adjusted estimate	Team performance adjusted estimate	Interpretation
Years of experience: 11–15 years vs. 1–5 years	Beta = −16.93 (95% CI: −33.21 to −0.64), *p* = 0.042	Not summarized as significant in the team-performance model	Lower adjusted meeting-effectiveness score for this group; interpret cautiously due to small reference category.
Organizational culture: high vs. very low	Beta = 20.06 (95% CI: 4.53 to 35.58), *p* = 0.012	beta = 0.63 (95% CI: 0.06 to 1.19), *p* = 0.030	Positive culture perception was independently associated with both outcomes.
Organizational culture: very high vs. very low	Beta = 21.60 (95% CI: 5.04 to 38.15), *p* = 0.011	beta = 0.71 (95% CI: 0.11 to 1.30), *p* = 0.020	The strongest adjusted pattern was observed among staff rating culture very highly.
Marital status	Not significant in adjusted model (*p* = 0.445–0.863)	Not summarized as significant	No consistent adjusted association.
Professional development	Not significant in adjusted model (*p* = 0.292–0.822)	Not significant in adjusted model (*p* = 0.906–0.911)	Strong bivariate pattern was attenuated after adjustment.
Resource access	Not significant in adjusted model (*p* = 0.133–0.446)	Not significant in adjusted model (*p* = 0.124–0.936)	Strong bivariate pattern was attenuated after adjustment.
Communication with leaders	Not significant in adjusted model (*p* = 0.674–0.960)	Not significant in adjusted model (*p* = 0.292–0.656)	Strong bivariate pattern may overlap with organizational culture perception.

Model 1 examined factors associated with meeting effectiveness score. Model 2 examined factors associated with team performance score. CI, confidence interval. The table summarizes statistically significant adjusted estimates and non-significant model domains to support the brief report format; detailed bivariate findings are provided as Supplementary material.

## Discussion

This study found a strong association between perceived staff meeting effectiveness and team performance among nurses and midwives. The findings highlight that routine meetings are not simply administrative activities but practical mechanisms through which communication, participation, and coordination occur. Well-structured meetings, with clear purpose, inclusive facilitation, and visible follow-up, may support stronger team functioning.

Organizational conditions appeared more influential than individual or logistical factors. While professional development, resources, leadership communication, and organizational culture were associated with outcomes in bivariate analyses, organizational culture remained the most consistent independent factor after adjustment. This suggests that meeting effectiveness is shaped by the broader work environment, including whether staff feel heard, supported, and able to contribute to meaningful change.

The finding on organizational culture is important for occupational health and public health management because culture is not only a background characteristic; it affects how teams communicate, escalate concerns, interpret leadership messages, and act on agreed priorities. A meeting agenda or template may improve structure, but it is unlikely to create value if staff do not believe that concerns will be taken seriously or that follow-up will occur. Conversely, a supportive culture can make routine meetings more credible by connecting discussion with action, learning, and accountability.

For healthcare managers, the practical implication is to treat meeting quality as a modifiable part of the operating system. First, recurring meetings should have a clear purpose, a standard agenda, explicit decision capture, and an action log with named owners and deadlines. Second, leaders should communicate before and after meetings, especially by closing the loop on issues raised by staff. Third, meeting improvement should be linked with workforce support, including facilitation skills, team communication, and escalation of recurrent resource constraints.

These actions are low cost and may be particularly useful in large hospitals with diverse workforces and heavy service demands. In such settings, managers may not be able to rapidly change staffing models, specialty structures, or workload pressures, but they can improve how existing forums are used. Better meetings may help staff understand priorities, coordinate across roles, and identify problems earlier. They may also support occupational wellbeing by reducing the frustration associated with repeated discussion without action.

The results should also be interpreted as a signal for targeted improvement rather than as proof that meetings alone drive performance. A practical next step for managers would be to combine brief meeting-effectiveness pulse checks with review of action-log completion, staff feedback on follow-up, and selected workforce or service indicators. This would allow organizations to move from perception-based assessment toward a more rounded understanding of whether meeting redesign translates into more reliable team action.

This study has limitations. The cross-sectional design does not allow causal inference; it cannot determine whether effective meetings improve team performance, whether stronger teams perceive meetings more positively, or whether both are influenced by wider organizational conditions. The study was conducted at a single hospital, which may limit transferability. Outcomes were self-reported and collected at one time point, creating possible social desirability bias and common-method variance. The study also did not directly observe meetings or assess objective indicators such as action-log completion, staff turnover, incident learning, or patient-safety outcomes. Some subgroup sizes were small, and residual confounding remains possible. Future studies should use longitudinal or intervention designs, include multiple sites, and link meeting quality with objective workforce and service outcomes.

In conclusion, staff meeting effectiveness and team performance were strongly associated and closely linked to modifiable organizational conditions. Routine meetings should therefore be managed as part of the healthcare work environment. Structured agendas, clear accountability, and consistent follow-up are practical, low-cost actions that may strengthen teamwork and service delivery, particularly in large and diverse healthcare settings.

## Data Availability

The data supporting the findings of this study are available from the corresponding author upon reasonable request, subject to institutional and ethical requirements.
