# Supplementary material for: Staff meeting effectiveness and team performance among nurses and midwives in a tertiary hospital in Qatar
Source: Front Psychol. 2026 Sep 23;17:1917610. doi: 10.3389/fpsyg.2026.1917610 (PMC13642206; doi:10.3389/fpsyg.2026.1917610)
Supplement: Supplementary file 1 [file Table_1.docx]

# Supplementary Table 1. Detailed bivariate findings summarized for peer review

This supplementary summary preserves details shortened from the Brief Research Report main text.

| **Variable** | **Summary** | **Meeting effectiveness p-value** | **Team performance p-value** |
| --- | --- | --- | --- |
| Age | Not significant | 0.150 | 0.670 |
| Gender | Not significant | 0.260 | 0.270 |
| Marital status | Significant for meeting effectiveness only | 0.035 | 0.058 |
| Educational status | Not significant | 0.400 | 0.660 |
| Years of nursing experience | Significant for meeting effectiveness only | 0.005 | 0.230 |
| Nationality region | Not significant | 0.210 | 0.330 |
| Shift type | Not significant | 0.570 | 0.540 |
| Average weekly hours | Borderline/non-significant for meeting effectiveness | 0.063 | 0.440 |
| Meeting frequency | Not significant | 0.830 | 0.330 |
| Meeting duration | Not significant | 0.590 | 0.770 |
| Meeting role | Not significant | 0.110 | 0.160 |
| Professional development | Significant for both outcomes | <0.001 | <0.001 |
| Resource access | Significant for both outcomes | <0.001 | <0.001 |
| Communication with leaders | Significant for both outcomes | <0.001 | <0.001 |
| Organizational culture perception | Significant for both outcomes | <0.001 | <0.001 |
